# Massive dispersal of *Coxiella burnetii* among cattle across the United States

**DOI:** 10.1099/mgen.0.000068

**Published:** 2016-08-25

**Authors:** Sonora Olivas, Heidie Hornstra, Rachael A. Priestley, Emily Kaufman, Crystal Hepp, Derek L. Sonderegger, Karthik Handady, Robert F. Massung, Paul Keim, Gilbert J. Kersh, Talima Pearson

**Affiliations:** ^1^​ Center for Microbial Genetics and Genomics, Northern Arizona University, PO Box 4073, Flagstaff, AZ 86011, USA; ^2^​ Rickettsial Zoonoses Branch, Centers for Disease Control and Prevention, 1600 Clifton Rd, Atlanta, GA 30333, USA; ^3^​ Informatics and Computing Program, Northern Arizona University, PO Box 5717, Flagstaff, AZ 86011, USA; ^4^​ Department of Mathematics and Statistics, Northern Arizona University, PO Box 5717, Flagstaff, AZ 86011, USA; ^5^​ Pathogen Genomics Division, Translational Genomics Research Institute, Phoenix, Arizona, USA

**Keywords:** Coxiella burnetii, Q fever, genotyping, dairy cattle, Phylogeography, SNP typing, canSNP

## Abstract

Q-fever is an underreported disease caused by the bacterium *Coxiella burnetii*, which is highly infectious and has the ability to disperse great distances. It is a completely clonal pathogen with low genetic diversity and requires whole-genome analysis to identify discriminating features among closely related isolates. *C. burnetii*, and in particular one genotype (ST20), is commonly found in cow’s milk across the entire dairy industry of the USA. This single genotype dominance is suggestive of host-specific adaptation, rapid dispersal and persistence within cattle. We used a comparative genomic approach to identify SNPs for high-resolution and high-throughput genotyping assays to better describe the dispersal of ST20 across the USA. We genotyped 507 ST20 cow milk samples and discovered three subgenotypes, all of which were present across the entire country and over the complete time period studied. Only one of these sub-genotypes was observed in a single dairy herd. The temporal and geographic distribution of these sub-genotypes is consistent with a model of large-scale, rapid, frequent and continuous dissemination on a continental scale. The distribution of subgenotypes is not consistent with wind-based dispersal alone, and it is likely that animal husbandry and transportation practices, including pooling of milk from multiple herds, have also shaped the patterns. On the scale of an entire country, there appear to be few barriers to rapid, frequent and large-scale dissemination of the ST20 subgenotypes.

## Data Summary

1. Genomes CMSC1, CMCA1c1, and ESFL1 have been deposited in GenBank; accession numbers: SRR3347458, SRR3347491 and SRR3347474: http://www.ncbi.nlm.nih.gov/Traces/sra/?run=SRR3347458;
  http://www.ncbi.nlm.nih.gov/Traces/sra/?run=SRR3347491;
  http://www.ncbi.nlm.nih.gov/Traces/sra/?run=SRR3347474;

## Impact Statement

Pathogen dissemination is typically limited by environmental survival, infectious dose, dispersal mechanisms and pathogen–host interactions. Understanding these characteristics is key for prevention and management of disease. *Coxiella burnetii*, the causative agent of Q fever in humans, survives well in the environment, is highly infectious, and readily dispersed via wind. *C. burnetii* ST20 is highly prevalent in cow’s milk, and even though it does not often cause human disease, it serves as a pragmatic model for understanding the dispersal of a pathogen with seemingly few, if any, dispersal barriers. We developed genotyping assays to subdivide this common genotype in an evolutionarily meaningful context. We then genotyped *C. burnetii* DNA extracted from cow milk collected through intensive longitudinal sampling and from processing facilities across the USA. Our results show that ST20 has persisted long enough for mutations to accumulate and that these variants are distributed across space and time. This is consistent with large-scale, rapid, frequent and continuous dissemination on a continental scale. Animal husbandry and transportation practices probably influence this distribution, which is not consistent with wind-based dispersal alone. At least for this clone of *C. burnetii*, there appear to be few dispersal barriers.

## Introduction

*Coxiella burnetii* is the etiological agent of the zoonotic disease Q fever. It causes an acute febrile illness that can result in pneumonia, hepatitis, and endocarditis in humans (Maurin & Raoult, 1999). It is endemic in ruminants yet difficult to detect as it often causes asymptomatic or subclinical disease; however, visible symptoms include abortion and premature delivery (Agerholm, 2013). *C. burnetii* is highly infectious and as few as one to ten viable cells can cause disease in humans (Benenson & Tigertt, 1956). The environment can become contaminated with *C. burnetii* when shed in feces, urine, milk and placental material from infected animals. Once in the environment, the bacteria persist in a spore-like form for extensive lengths of time, even under non-ideal conditions (Kersh**et al.**, 2013; Scott & Williams, 1990). *C. burnetii* is easily aerosolized and can be transferred by wind over long distances. In 1998, 147 cases of Q fever were reported in Birmingham, UK and tied to outdoor lambing at a single farm and dispersal by unusually strong winds (Hawker**et al.**, 1998). While attack rates decreased with distance from the source farm, the main cluster of cases stretched as far away as 18 km (Hawker**et al.**, 1998). Examples of aerosol-based transmission and dispersal of *C. burnetii* are common (Nusinovici**et al.**, 2015; Tissot-Dupont**et al.**, 2004). High infectivity, environmental persistence, and ease of aerosolization all have important implications for dispersal of this pathogen and pose the question of whether there are any barriers that may limit or slow its dissemination.

*C. burnetii* is distributed across the world (with the possible exception of New Zealand), however geographic associations are limited to a few individual genotypes that seem to coexist with widely distributed genotypes (D'Amato**et al.**, 2016). The widespread distribution of most genotypes may be due to an almost complete lack of barriers to dissemination that can result in frequent, widespread and rapid dispersion that prevents such geographic associations. Unfortunately, studies exploring mechanisms and patterns of geographic spread are limited. Genotyping studies involving isolates collected around the world show that many genotypes have a widespread geographic distribution (Glazunova *et al.*, 2005; Hornstra *et al.*, 2011). Many regional genotyping studies have employed multiple locus VNTR analysis (MLVA) (Arricau-Bouvery *et al.*, 2006; Svraka *et al.*, 2006), however this method is not high-throughput, inter-lab comparisons using MLVA data can be problematic and the lack of a standardized MLVA scheme further complicates comparisons of results (Sidi-Boumedine *et al.*, 2015). Using PCR-based assays (Hornstra *et al.*, 2011) to detect single-nucleotide polymorphisms (SNPs) that are diagnostic for clades defined by multispacer sequence typing (MST) (Glazunova *et al.*, 2005). Pearson *et al.* (2014) and Bauer *et al.* (2015) conducted high-throughput surveys of genotypes associated with commercial milk across the USA and within the state of Indiana, USA, respectively. These studies showed that ST20 and ST8 were common across the USA and more likely to be associated with cows and goats, respectively (Bauer *et al.*, 2015; Pearson *et al.*, 2014). Together, these results indicate that if geographic associations exist, higher resolution genotyping coupled with intensive sampling is needed to detect it. To test this hypotheses on *C. burnetii* dispersal, we revisit the ST20 samples characterized by Pearson *et al.* (2014), include additional ST20 samples and, importantly, use higher resolution genotyping assays to search for geographic and temporal associations. The higher resolution is possible due to additional genomic sequences from ST20 isolates.

## Methods

Multi-locus variable number tandem repeat analysis (MLVA) has shown higher levels of resolution than multispacer sequence typing (MST) (Glazunova**et al.**, 2005) and even variation within ST20 (Astobiza**et al.**, 2012). However, MLVA is not well-suited for genotyping the low levels of *C. burnetii* DNA contained in complex backgrounds that are recovered from environmental and animal sampling. Most importantly, it is not clear how MLVA genotypes are related to each other, further limiting the phylogenetic utility of MLVA in understanding dispersal patterns. Single nucleotide polymorphisms (SNPs) are ideal for accurately depicting evolutionary relationships among clonal bacteria (Pearson**et al.**, 2004, 2009), including *C. burnetii* (Pearson**et al.**, 2013). Furthermore, assays based on SNP signatures are ideal for low-level genotyping of *C. burnetii* (Pearson**et al.**, 2014). However, comparisons of closely-related whole genome sequences are required to find the rare SNP signatures capable of high-resolution discrimination (Eppinger**et al.**, 2014; Scholz**et al.**, 2014), and phylogenetic discovery bias (Pearson**et al.**, 2004) must be taken into account when SNP loci are characterized in samples whose genomes are not sequenced. We therefore sequenced and compared three *C. burnetii* isolates from the USA and belonging to ST20 to find SNPs, developed genotyping assays to target those SNPs, and tested the assays against the 507 ST20 samples from our previous study (Pearson**et al.**, 2014). In addition, we included an extension of the time series presented there.

### Culturing and DNA isolation for whole-genome sequencing.

Three *C. burnetii* isolates (CMSC1, CMCA1c1 and ESFL1) were grown axenically in Acidified Citrate Cysteine Medium 2 (ACCM2) following the methods of Omsland**et al.** (2011). DNA was extracted and purified using the QIAamp DNA mini kit tissue protocol (Qiagen) according to manufacturer directions.

Two of these isolates were derived from unpasteurized cow’s milk purchased in South Carolina (CMSC1) and California (CMCA1c1) in 2007. The third isolate, ESFL1, was from an environmental sample collected from soil at a cow dairy in June 2008 in Florida.

### Whole-genome sequencing and SNP detection.

DNA libraries were prepared for multiplexed, paired-end sequencing on GAIIx sequencing platforms (Illumina). Sequence data generated have been submitted to the Sequence Read Archive (CMSC1, SRR3347458; CMCA1c1, SRR3347491; and ESFL1, SRR3347474). SNP detection among the three whole-genome sequences presented here and the three whole-genome sequences of ST20 from NCBI (Table S1, available in the online Supplementry Material) was performed using the Northern Arizona SNP Pipeline (NASP) (http://tgennorth.github.io/NASP/) and the *In Silico* Genotyper pipeline (Sahl**et al.**, 2015) with RSA 493 (NC_002971.3) as the reference. All SNPs (excluding those that were autapomorphic for the reference) are given in Table S2 available in the online supplementary material.

### Phylogenetics of ST20 isolates.

We used parsimony criteria and a heuristic search with default options using PAUP 4.0b10 (Swofford, 2003). As a measure of accuracy, we report homoplasies using the consistency index (Archie, 1996); bootstrapping is a poor method for measuring accuracy for trees with little homoplasy (Felsenstein, 1985) such are recovered when using SNPs from clonal organisms like *C. burnetii* (Pearson**et al.**, 2013). The phylogeny was rooted using RSA493 which was previously established as an outgroup to ST20 (Pearson**et al.**, 2013). Phylogenetic branches were named according to precedent (Hornstra**et al.**, 2011), assigned to SNPs (Table S2), and designated on trees (Fig. S1, available in the online Supplementary Material). In short, each branch contains a prefix ‘Br.III’ to indicate the genomic group, followed by an assigned number based upon the order of branch discovery within the clade. For SNPs that are specific to a single genome, the genome name, rather than a branch number is used. This method maintains the branch name from previous publications and allows for the identification of novel branches. These SNPs (Table S2) can therefore serve as a resource for others looking to differentiate among isolates in this clade. Novel genome sequences will cause existing branches to be split, requiring additional branch names and updating the branch designation of these SNPs. For evolutionarily stable characters such as SNPs found in clonal organisms like *C. burnetii*, a single locus can define a branch and thus serve as a ‘canonical SNP’ (Hornstra**et al.**, 2011). As such, the character states of only a small number of SNP loci need to be interrogated in order to place an unknown strain into the established phylogenetic order.

### Assay development for increased resolution of ST20.

**Fig. 1.**
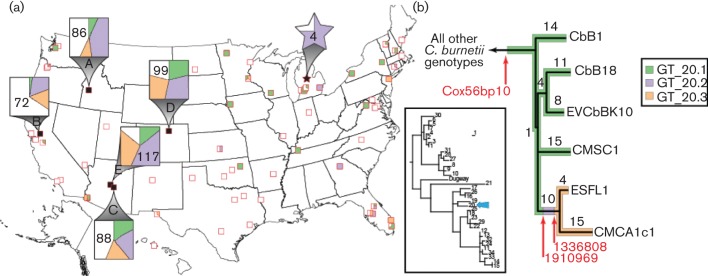
Phylogeography of samples. (a) Map shows the location of the sampled bovine herd from Michigan (star), and milk processing plants (squares) that produced samples with ST20 genotypes. Large squares indicate longitudinally sampled processing plants. Shapes include a pie chart to indicate the distribution of ST20 subgenotypes. White portions of pie charts indicate ST20, but also that a subgenotype of ST20 could not be established. (b) Phylogenetic tree depicting known MST genotypes and the location of ST20 (inset). An expanded phylogeny of ST20 shows the location of samples with available genome sequences, the number of SNPs on each branch (Table S2), the location of assays (red arrows), and a color designation for each subgenotype. This maximum-parsimony tree was reconstructed using 82 SNPs from whole-genome comparisons of six ST20 genomes and RSA493 (used to root the tree).

TaqMan® minor groove binding (MGB) non-fluorescent quencher (NFQ) dual-probe genotyping assays (Supplementary Appendix A) were designed for SNPs on the branch connecting two of the three genomes from the isolates from the USA. This branch was targeted as the other genomes were not available at the beginning of this study. These assays allowed for the potential division of U.S. ST20 samples into three sub-ST20 genotypes (Fig. 1b). Both TaqMan® assays were designed using Primer Express® Software v3.0.1 and optimized through rigorous testing of the limits of each assay (Supplementary Appendix A).

### Subgenotyping of ST20 derived from bulk tank milk

DNA extracted from bulk tank milk samples (*n* = 507) from processing plants across the USA, previously identified as positive for *C. burnetii* DNA, and genotyped as ST20 (Pearson**et al.**, 2014) were screened across both the Cox1910969 and Cox1336818 assays developed here (Supplementary Appendix A). DNA was tested in duplicate to ensure consistency of results. Cox1910969 assay conditions were as follows: 1 µl of DNA was added to form a total reaction volume of 10 µl that contained 5 µl of 2× TaqMan® Universal PCR Master Mix (Life Technologies), 0.20 µl of each 20 µM primer, 0.10 µl of the 20 µM FAM MGB-NFQ probe, 0.15 µl of the 20 µM VIC MGB-NFQ probe, and 3.35 µl of sterile, molecular-grade water. Cox1336818 reaction conditions were identical to the above but with the following exceptions: 0.06 µl of each 20 µM MGB-NFQ probe and 3.48 µl of sterile, molecular-grade water were added. No-template-controls were tested on each PCR plate to allow the detection of cross-contamination during DNA template addition. Positive template controls of whole-genome amplified DNA samples that were known to be of the ancestral allele state and the derived allele state for both assays were also tested on all PCR plates to detect assay or instrument errors. Thermal cycling conditions for both assays were as follows: 50 °C for 2 min., 95 °C for 10 min., followed by 45 cycles of 95 °C for 15 sec. and 60 °C for 1 min. PCR was performed on a 7900HT Fast real-time PCR system (Applied Biosystems) with SDS v2.4 software. Allele calls were made based upon amplification of competitive probes, with a manual threshold of 0.1 and an automatic baseline. The derived allele state for Cox1336818 (T), is indicative of the presence of genotype 20.3. The ancestral allele for Cox1910969 (C), is indicative of genotype 20.1. Samples that show the ancestral allele for Cox1336818 (C) and the derived allele for Cox1910969 (T) contain genotype 20.2 (Fig. 1b). For either assay, the amplification of both probes in a given sample is typically evidence of a mixture (see Supplementary Appendix A). The amplification of both probes in both assays is indicative of the presence of all three genotypes in a single sample. The amplification of both probes for one assay and a single probe for the other assay is indicative of two genotypes. Given the low amounts of *C. burnetii* DNA in some samples, we expected some assays to fail. When both genotyping assays failed, we were not able to determine which subgenotype(s) within ST20 was present. The failure of one assay did not preclude our ability to determine subgenotype(s) as a derived call for Cox1336818 is indicative of genotype 20.3. Similarly, an ancestral call for Cox1910969 is indicative of genotype 20.1. Both assays needed to amplify to identify the presence of genotype 20.2. The presence of an undetermined genotype(s) was noted. In some cases, the amplification of both probes for one assay and a failure of the other assay is indicative of one genotype as well as an undetermined genotype.

### Geographic and temporal distribution of ST20 subgenotypes in bovine milk.

All subgenotype results were compiled and represented on a map of the USA (Fig. 1a) to depict the geographic distribution of ST20 subgenotypes. In addition, we graphed all subgenotype results for the five milk processing plants that were sampled at least monthly from 2009 to 2014 as a function of time in order to determine whether there was a temporal distribution of ST20 subgenotypes among processing plants.

For all samples, we tested for any geographic structuring among four quadrants of the country (northeast, southeast, southwest and northwest) using a Chi-squared test as implemented in the R base package (chisq.test), and post-hoc tests using the fdr method as implemented in the R package fifer (chisq.post.hoc). We also performed statistical tests for the correlation between presence of subgenotypes and of geographic and temporal structuring of the five longitudinally sampled facilities. We tested the correlation of the presence of the subgenotype in two ways: we performed a log-linear analysis (Agresti & Kateri, 2011) and, to verify the result, we also performed a non-parametric permutation test on the determinant correlation matrix (a determinant near one represents uncorrelated variables).

To explore the temporal structure, we analyzed each subgenotype separately and fit generalized additive models (Wood, 2006) using a cyclic cubic regression spline with a within-year smoothed term for each milk processing plant. Statistical significance was assessed via likelihood ratio test statistics compared with the asymptotic *χ*^2^ distribution. To account for analyzing each subgenotype separately, we adjust the *P*-values using a Bonferroni adjustment factor of three.

## Results

### Phylogenetics of current ST20 isolates

The three European ST20 isolates from NCBI were all isolated from bovine placental material or a bovine vaginal swabs and group separately from genomes from the USA (Fig. 1b). Excluding the SNPs that were autapomorphic for the reference strain, there were a total of 52 SNPs. Maximum-parsimony analyses yielded a single phylogenetic tree with a consistency index of 1.000 as no SNPs were homoplastic.

### ST20 subgenotypes are ubiquitious in bulk milk samples throughout the USA

The locations indicated by the expanded squares in Fig. 1(a) were sampled at least monthly from May 2010 to July 2014 by purchasing milk at local stores that had been processed in these locations as indicated by the plant code on each bottle. The smaller squares on the map indicate milk processing plants sampled to assess geographic distribution of *C. burnetii* genotypes (Fig. 1a) (Pearson**et al.**, 2014). The expanded star indicates the location of a single farm in Michigan from which the bulk milk tank and individual animals were sampled according to the methods of Pearson**et al.** (2014). The assays used here on ST20 samples allow for the detection of three distinct ST20 subgenotypes. Of the 507 samples, 95 samples contained genotype 1 (GT_20.1), 141 samples contained genotype 2 (GT_20.2), 103 samples contained genotype 3 (GT_20.3), 55 samples contained more than one genotype, and 236 samples contained an undetermined genotype(s), meaning that they were ST20 but the presence of an exact subgenotype could not be determined if one or both of our assays failed. It is important to note that in many instances, the subgenotype could be determined even if one of the assays failed. For example, the derived allele state for Cox1336818 (T), is indicative of the presence of genotype 20.3, independent of the results, or lack thereof for Cox1910969. These failures were likely to be due to low levels of target DNA. All three subgenotypes were found across the USA (Fig. 1a) and some processing plants contained more than one genotype. There were statistically significant differences in the composition of subgenotypes across different regions of the USA [χ^*2*^ (6, *n*=674)=46.77, *P.adj* <0.001]. The genotype composition sampled from the northwest was significantly different from the other three regions (*P.adj <*0.001), driven by the high proportion of genotype 20.2 from the single processing plant A. This is consistent with comparisons of only the longitudinally sampled facilities which also suggest that milk from different processing plants differs in genotype compositions (see below).

### ST20 subgenotypes are present through time in bulk tank milk in the USA

**Fig. 2.**
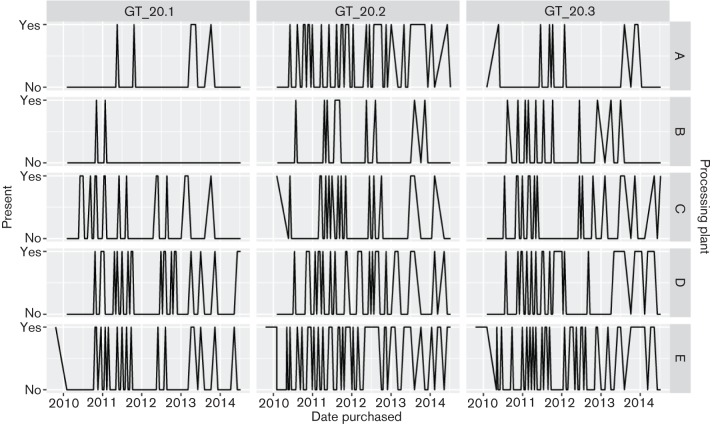
Longitudinal presence and absence of subgenotypes across processing plants.

The presence or absence of each genotype over time across five longitudinally sampled processing plants show a strong difference in percentage presence between plants but little temporal structure (Fig. 2). These data also show differences by processing facility for each subgenotype: genotype 20.1 [χ*^2^* (4, *n*=182)=17.69, *P.adj* <0.01], genotype 20.2 [χ*^2^* (4, *n*=286)=23.46, *P.adj* <0.01], genotype 20.3 [χ*^2^* (4, *n*=206)= 24.33, *P.adj* <0.01). Although the prevalence of each subgenotype differed by processing plant, there was little readily observable geographic pattern. For example, the genotype proportions from the two plants in Arizona differed despite close geographic proximity. The presence or absence of one genotype does not provide any information about the presence or absence of another genotype. We found no significant correlation between subgenotype presence and absence among any of the five processing plants sampled between 2009 and 2014 (permutation test: *det(R)*=0.99, *P*=0.48; log-linear test: [χ*^2^* (3, *n*=398)=4.66, *P.adj* =0.20]. Results of the temporal analysis show no significant yearly trends with the single exception of an August peak for processing facility ‘A’ for genotype 20.2 [χ*^2^* (8, *n*=398)=8.383, *P.adj* =0.0205]. The statistically significant differences among processing facilities and temporal trends explained a maximum of 17 % deviance and thus are likely to have little biological significance at this level of phylogenetic and geographic resolution.

### Only a single ST20 subgenotype was found on a single farm at one time point

In a single herd in Michigan, genotype 20.1 was detected in a sample from a single cow and in two samples from the bulk milk tank.

## Discussion

*Coxiella burnetii* is prevalent around the world. It is endemic in ruminants and contaminates the environment through shedding in urine, milk, fecal and placental material. It has the ability to persist in the environment without degradation for long periods of time, is easily aerosolized, and can be transmitted over a few kilometers. ST20 is highly prevalent in cow’s milk across the USA (Pearson**et al.**, 2014), and even though it is rarely associated with human disease, its high prevalence makes it a pragmatic model for better understanding of how this species is dispersed, persists and perhaps could be controlled.

The whole genome SNP phylogeny of three isolates from the USA and three European isolates of ST20 shows that members of this clade have persisted long enough for SNP mutations to arise. The basal position of CbB1 (France) and the derived position of the ESFL1/CMCA1 clade suggest that ST20 may have arisen in Europe and subsequently spread to North America. The short branch (1 SNP) that separates the CbB1 lineage from the other genomes and the subsequent polytomy leading to a European clade (CbB18/EVCbBK10) and two groups in the USA is indicative of a radiation that may indicate a rapid dissemination from Europe to the USA where multiple lineages became ecologically established. While these hypotheses are consistent with the data generated from the SNP-based assays presented here, more SNP loci and more genomes and samples collected from Europe and other regions need to be tested to confirm or refute this global model of ST20 evolution.

To best illustrate dissemination patterns and phylogeographic structuring, high-throughput whole-genome sequencing of these DNA from milk samples would provide the best estimate of circulating diversity and relationships among samples. Unfortunately, current sequencing technologies are not capable of providing adequate genome coverage for environmental samples containing low levels of target DNA in complex mixtures. However, sequencing a small number of isolates and comparing their genomes can lead to the discovery of SNPs that can be converted into high-throughput and highly sensitive assays that are amenable to environmental samples. This approach can provide additional resolution and phylogenetic insights into the behavior of a microbial population that may otherwise be difficult to study (Pearson**et al.**, 2004, 2009). The two new ST20 assays presented here (Cox1910969 and Cox1336818) provide increased phylogenetic resolution of the ubiquitous ST20 genotype of *C. burnetii* that dominates dairy bovine populations in the USA and appears to be common throughout the world. These two assays, in conjunction with the ST20 assay (Cox56bp10) allow for the segregation of three ST20 genotypes. The future development of additional assays based upon other SNPs within this phylogeny (Table S2) will provide further insights into the diversity of *C. burnetii* in commercial bovine milk in the USA and the presence or absence of geographic segregation within any of these ST20 genotypes. Although we could not identify SNPs capable of differentiating the European and American isolates that were sequenced, it is possible that European ST20 samples are distinct from samples from the USA, however, SNPs that provide resolution within GT_20.1 (Table S2) would need to be assayed against samples from the USA and Europe, the latter of which were not available for the current study. If such phylogeographic structuring exists and GT_20.1 is dominant in Europe, the assays presented here would not provide additional resolution among European isolates. Conversely, the lack of a European-specific ST20 subclade would also be indicative of massive dissemination and the lack of dispersal obstacles on a global scale. If this were the case, genotypes 20.2 and 20.3 would be expected among European samples.

Pearson**et al.** (2014) proposed a model of rapid spread and subsequent persistence of ST20 in the USA. While dispersal of small-cell variants in a quiescent phase can occur over long periods of time with no evolutionary change, the widespread distribution of ST20 would have required massive amplification by animal hosts. Mutation rate estimates are not currently available for *C. burnetii*, limiting our ability to quantify dispersal speed, however as ST20 was not found in the USA prior to 2007 and previous samples from cattle in 1947 and 1958 were a different ST (Hornstra**et al.**, 2011), this dispersal must have occurred in less than approximately 50 years (Pearson**et al.**, 2014). Our characterization here of three subgenotypes of ST20 dispersed across the USA and across time is consistent with this model but adds elements of dispersal frequency and pathogen population size. The coexistence of multiple subgenotypes of ST20 across the country demonstrates a general lack of geographic structuring at the country scale, although genotype compositional differences do exist (Fig. 1a). That we found only one subgenotype within a single herd may be suggestive of spatial structure at the herd level, however. The genotype proportions among the longitudinally sampled facilities do show a correlation with facility, however the extent to which this pattern is geographically related (rather than facility-related) appears to be small. Similarly, the temporal coexistence of ST20 subgenotypes suggests the lack of temporal patterns. Frequent convergent and independent evolution of genotypes provides an alternative mechanism by which these three subgenotypes could be widespread with structured populations. However, convergent evolution is rare in *C. burnetii* (Hornstra**et al.**, 2011; Pearson**et al.**, 2013) and is non-existent here, given the complete lack of homoplasy among the ST20 genomes studied. While these three subgenotypes show little, if any, geographic or temporal structure across the USA, our results do not preclude the likelihood that such structure may exist within a subgenotype or within a herd as our results from the single dairy farm in Michigan suggest.

In any case, our results provide evidence of rapid, frequent and continuous dissemination on almost a continental scale. The distribution patterns of genotypes, coupled with the high prevalence of ST20 throughout the country, is consistent with a lack of genetic bottlenecks and the likely involvement of a large portion of the pathogen population in dispersal. The coexistence of multiple subgenotypes and the persistence of this coexistence through time is also consistent with this model of continuous, population-wide dispersal and not due to ongoing adaptation within ST20 subtypes. In addition, coexistence of subgenotypes demonstrates the ability for novel types to become established and persist with other subgenotypes. The speed, frequency and extent of population-wide involvement in dispersal would be even better defined by further phylogenetic resolution and quantification of mutation rate to define the speed of dispersal.

Geographic or temporal patterns may exist at the level of an individual herd. Such structure would be largely undetectable with these samples as they contain mixtures of milk from an unknown number of separate farms. Our detection of only one genotype in a single bovine herd sampled from Michigan (Fig. 1a) suggests the possibility of population structuring at the herd level. Nonetheless, our results suggest that continual, frequent and widespread movement of variants has resulted in massive dispersal of ST20 on a continental scale.

The mechanisms driving these regional and potentially global patterns of dissemination and persistence are not known and difficult to imagine. However, they are likely to involve animal husbandry and transportation practices coupled with wind dispersal and long-term environmental viability of *C. burnetii*. The degree to which ST20 in other regions and other sequence types share these dispersal characteristics is unknown, however some genotypes have only been reported in certain countries (D'Amato**et al.**, 2016). Certain genotypes of *C. burnetii* (ST20 and ST8) are thought to be evolutionarily adapted to cows and goats, respectively (Pearson**et al.**, 2014). The extent to which other genotypes are host-adapted and the propensity for host movement may play a large role in defining the dispersal of each genotype as well as the ability for different genotypes to coexist. Efficient and effective control efforts for this pathogen must be based upon a thorough understanding of the mechanisms of local, regional and global dispersal and persistence of different genotypes.

## Supplementary Data

1733 Supplementary File 1
