# Supplementary material for: Massive dispersal of Coxiella burnetii among cattle across the United States
Source: Microb Genom. 2016 Aug 25;2(8):e000068. doi: 10.1099/mgen.0.000068 (PMC5320587; doi:10.1099/mgen.0.000068)
Supplement: Supplementary file 1 [file mgen-02-68-s001.pdf]

Supplemental Table 1: External genomes

Strain name	NCBI ID number	Accession number	SRA number	Country of origin	Isolation year	Isolation source	References
NMI_RSA493	2603970	SAMN02603970	n/a	United States	1935	*Dermacentor andersoni*	Seshadri, R. et al. (2003).
CbB1	2759516	SAMEA2377214	ERS407827	France	Unknown	Bovine Plancental Cotyledon	Sidi-Boumedine K. et al. (2014).
CbB18	2759519	SAMEA2377215	ERS407828	Denmark	2008	Bovine Plancental Cotyledon	Sidi-Boumedine K. et al. (2014).
EVCbBK10	2759517	SAMEA2377218	ERS407831	Sweden	2010	Bovine Vaginal Swab	Sidi-Boumedine K. et al. (2014).

Supplemental Table 2: SNP matrix

Ref	Contig	Position	RSA493	CbB1	CbB18	EVCbBK10	CMSC1	ESFL1	CMCA1	Branch Name
NC_004704.1	29648114	15229	G	G	G	G	G	G	A	Br.III.CMCA1
NC_002971.3	77358712	1715	C	C	C	C	C	C	T	Br.III.CMCA1
NC_002971.3	77358712	43206	G	G	G	G	G	G	A	Br.III.CMCA1
NC_002971.3	77358712	105893	A	A	A	A	A	A	T	Br.III.CMCA1
NC_002971.3	77358712	244314	G	G	G	G	G	G	A	Br.III.CMCA1
NC_002971.3	77358712	289063	C	C	C	C	C	C	T	Br.III.CMCA1
NC_002971.3	77358712	319511	G	G	G	G	G	G	A	Br.III.CMCA1
NC_002971.3	77358712	363985	C	C	C	C	C	C	T	Br.III.CMCA1
NC_002971.3	77358712	390321	G	G	G	G	G	G	A	Br.III.CMCA1
NC_002971.3	77358712	443249	C	C	C	C	C	C	T	Br.III.CMCA1
NC_002971.3	77358712	920619	A	A	A	A	A	A	T	Br.III.CMCA1
NC_002971.3	77358712	948674	C	C	C	C	C	C	T	Br.III.CMCA1
NC_002971.3	77358712	1076230	C	C	C	C	C	C	T	Br.III.CMCA1
NC_002971.3	77358712	1156701	G	G	G	G	G	G	A	Br.III.CMCA1
NC_002971.3	77358712	1273112	G	G	G	G	G	G	A	Br.III.CMCA1
NC_002971.3	77358712	10548	C	C	C	C	T	C	C	Br.III.CMSC1
NC_002971.3	77358712	38100	C	C	C	C	T	C	C	Br.III.CMSC1
NC_002971.3	77358712	87674	C	C	C	C	T	C	C	Br.III.CMSC1
NC_002971.3	77358712	105902	A	A	A	A	G	A	A	Br.III.CMSC1
NC_002971.3	77358712	111833	G	G	G	G	A	G	G	Br.III.CMSC1
NC_002971.3	77358712	597364	G	G	G	G	A	G	G	Br.III.CMSC1
NC_002971.3	77358712	649128	C	C	C	C	T	C	C	Br.III.CMSC1
NC_002971.3	77358712	840079	C	C	C	C	T	C	C	Br.III.CMSC1
NC_002971.3	77358712	988731	G	G	G	G	A	G	G	Br.III.CMSC1
NC_002971.3	77358712	1029226	G	G	G	G	A	G	G	Br.III.CMSC1
NC_002971.3	77358712	1337787	G	G	G	G	A	G	G	Br.III.CMSC1
NC_002971.3	77358712	1496838	G	G	G	G	A	G	G	Br.III.CMSC1
NC_002971.3	77358712	1561439	G	G	G	G	A	G	G	Br.III.CMSC1
NC_002971.3	77358712	1730171	G	G	G	G	A	G	G	Br.III.CMSC1
NC_002971.3	77358712	1807175	G	G	G	G	A	G	G	Br.III.CMSC1
NC_002971.3	77358712	42329	C	C	T	T	C	C	C	Br.III.004
NC_002971.3	77358712	698163	G	G	A	A	G	G	G	Br.III.004
NC_002971.3	77358712	952355	T	T	A	A	T	T	T	Br.III.004
NC_002971.3	77358712	1790016	C	C	T	T	C	C	C	Br.III.004
NC_002971.3	77358712	46914	T	T	C	T	T	T	T	Br.III.CbB18
NC_002971.3	77358712	70046	G	G	A	G	G	G	G	Br.III.CbB18
NC_002971.3	77358712	564575	T	T	C	T	T	T	T	Br.III.CbB18
NC_002971.3	77358712	803805	T	T	C	T	T	T	T	Br.III.CbB18
NC_002971.3	77358712	1022346	G	G	A	G	G	G	G	Br.III.CbB18
NC_002971.3	77358712	1074196	C	C	T	C	C	C	C	Br.III.CbB18
NC_002971.3	77358712	1291059	G	G	A	G	G	G	G	Br.III.CbB18
NC_002971.3	77358712	1343194	A	A	G	A	A	A	A	Br.III.CbB18
NC_002971.3	77358712	1523454	G	G	A	G	G	G	G	Br.III.CbB18
NC_002971.3	77358712	1602030	A	A	G	A	A	A	A	Br.III.CbB18
NC_002971.3	77358712	1886713	G	G	A	G	G	G	G	Br.III.CbB18
NC_002971.3	77358712	52641	C	C	C	T	C	C	C	Br.III.EVCbBK10
NC_002971.3	77358712	138516	G	G	G	A	G	G	G	Br.III.EVCbBK10
NC_002971.3	77358712	208520	C	C	C	T	C	C	C	Br.III.EVCbBK10
NC_002971.3	77358712	229301	T	T	T	G	T	T	T	Br.III.EVCbBK10
NC_002971.3	77358712	439157	C	C	C	T	C	C	C	Br.III.EVCbBK10
NC_002971.3	77358712	826545	T	T	T	C	T	T	T	Br.III.EVCbBK10
NC_002971.3	77358712	1186826	G	G	G	A	G	G	G	Br.III.EVCbBK10
NC_002971.3	77358712	1465623	T	T	T	C	T	T	T	Br.III.EVCbBK10

NC_002971.3	77358712	121018	C	T	C	C	C	C	C	Br.III.CbB1
NC_002971.3	77358712	497338	A	G	A	A	A	A	A	Br.III.CbB1
NC_002971.3	77358712	529131	G	A	G	G	G	G	G	Br.III.CbB1
NC_002971.3	77358712	589646	C	T	C	C	C	C	C	Br.III.CbB1
NC_002971.3	77358712	724186	C	T	C	C	C	C	C	Br.III.CbB1
NC_002971.3	77358712	872847	C	T	C	C	C	C	C	Br.III.CbB1
NC_002971.3	77358712	1077341	G	A	G	G	G	G	G	Br.III.CbB1
NC_002971.3	77358712	1143646	C	T	C	C	C	C	C	Br.III.CbB1
NC_002971.3	77358712	1516232	G	A	G	G	G	G	G	Br.III.CbB1
NC_002971.3	77358712	1562356	G	A	G	G	G	G	G	Br.III.CbB1
NC_002971.3	77358712	1697393	G	A	G	G	G	G	G	Br.III.CbB1
NC_002971.3	77358712	1729026	T	C	T	T	T	T	T	Br.III.CbB1
NC_002971.3	77358712	1868714	T	C	T	T	T	T	T	Br.III.CbB1
NC_002971.3	77358712	1971982	G	A	G	G	G	G	G	Br.III.CbB1
NC_002971.3	77358712	130935	A	A	G	G	G	G	G	Br.III.005
NC_002971.3	77358712	665216	T	T	T	T	T	C	T	Br.III.ESFL1
NC_002971.3	77358712	885919	G	G	G	G	G	C	G	Br.III.ESFL1
NC_002971.3	77358712	971145	G	G	G	G	G	A	G	Br.III.ESFL1
NC_002971.3	77358712	1268947	G	G	G	G	G	A	G	Br.III.ESFL1
NC_002971.3	77358712	722599	T	T	T	T	T	A	A	Br.III.006
NC_002971.3	77358712	955680	G	G	G	G	G	A	A	Br.III.006
NC_002971.3	77358712	1055273	T	T	T	T	T	C	C	Br.III.006
NC_002971.3	77358712	1079080	G	G	G	G	G	A	A	Br.III.006
NC_002971.3	77358712	1336818	C	C	C	C	C	T	T	Br.III.006
NC_002971.3	77358712	1381152	G	G	G	G	G	A	A	Br.III.006
NC_002971.3	77358712	1556295	T	T	T	T	T	C	C	Br.III.006
NC_002971.3	77358712	1565238	G	G	G	G	G	A	A	Br.III.006
NC_002971.3	77358712	1910969	C	C	C	C	C	T	T	Br.III.006
NC_002971.3	77358712	1911235	G	G	G	G	G	A	A	Br.III.006

Supplemental Fig. 1: Branch designations

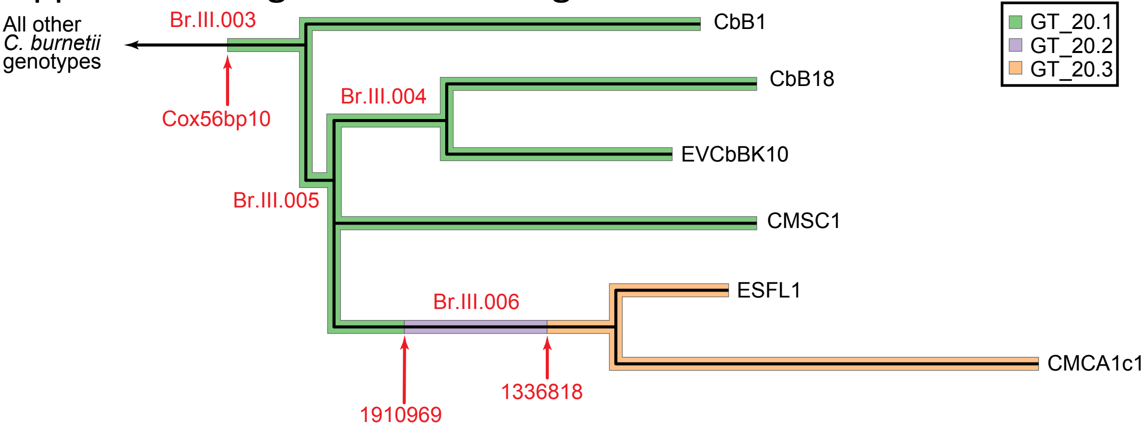

Appendix A: Assay information: validation and testing for Cox1336818 and Cox1910969

Summary

The purpose of this document is to report on the validation and testing of two TaqMan assays, Cox1336818 and Cox1910969, designed for subgenotyping of *Coxiella burnetii* ST20 genotypes. These assays (Table 1) provide additional resolution among samples of *C. burnetii* that are sequence type 20 (ST20) [1-3]. Both assays are located on the branch separating the genomes ESFL1 and CMCA1c1 from all other ST20s as well as all other STs of *Coxiella* (Figure 1). We tested the following parameters: precision, limits of quantitation, linearity, selectivity, limits of detection, and robustness. In addition, we compared the ΔCt values between the assays presented here, which target a single copy per genome, to the ΔCt values from an IS1111 assay [4], which targets multiple copies per genome and is used for detecting *C. burnetii*. The goal being to determine at what ΔCt values for IS1111 we see negative amplification with our single-target assays.

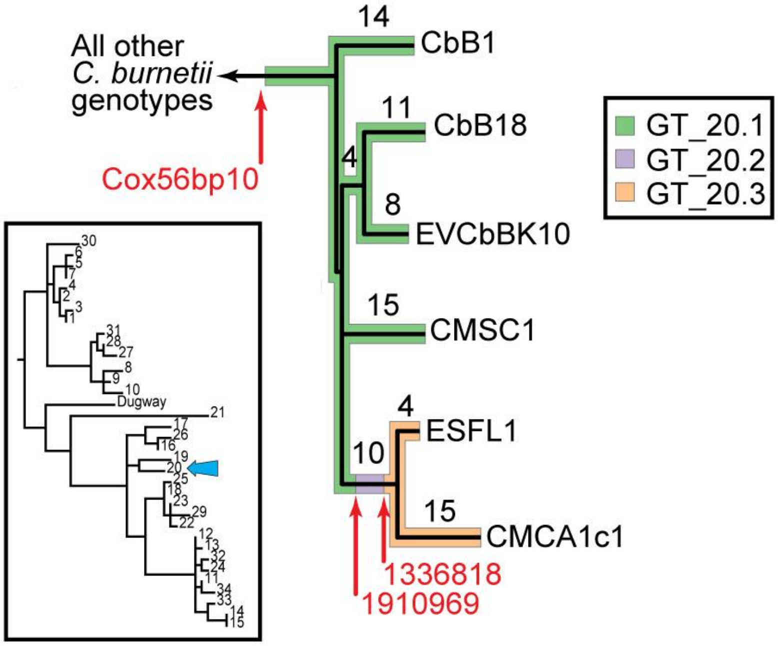

Figure 1: Phylogenetic tree indicating MST genotypes drawn according to Hornstra et al. [2] and rooted according to Pearson et al. [3] (boxed). The blue arrow indicates the location of ST20 in relation to all other sequence types of *Coxiella*. Enlarged phylogenetic tree for ST20 genomes only is shown. Location of the assays used in this study are indicated; subgenotypes described in the manuscript are indicated by colors on branches. The ancestral to derived SNP change for both assays is C/T.

PCR Conditions

Unless otherwise noted in a specific validation test, the following conditions were used for the Cox1910969 assay: 1 µL of DNA was added to form a total reaction volume of 10 µL that contained 5 µL of 2x TaqMan® Universal PCR Master Mix (Life Technologies, CA, USA; p/n 4304437), 0.20 µL of each 20 µM primer (Table 1), 0.10 µL of the 20 µM FAM MGB-NFQ probe (Table 1), 0.15 µL of the 20 µM VIC MGB-NFQ probe (Table 1), and 3.35 µL of sterile, molecular grade water. For the Cox1336818 assay, reaction conditions were identical to those above but with the following exceptions: 0.06 µL of each 20 µM MGB-NFQ probe (Table 1) and 3.48 µL of sterile, molecular grade water were added. Unless otherwise specified in this document, thermal cycling conditions for both assays were as follows: 50°C for 2 min., 95°C for 10 min., followed by 45 cycles of 95°C for 15 sec., and 60°C for 1 min. PCR was performed on an Applied Biosystems 7900HT Fast real-time PCR system with SDS v2.4 software. All data were analyzed using SDS v2.4 software with a manual threshold of 0.1 and an automatic baseline.

Table 1: Primers and Probes for the Cox1336818 and Cox1910969 assays.

Assay Name	Primer and Probe Names	Primer and Probe Sequences 5' → 3'	SNP State Ancestral/Derived
Cox1336818	Cb1336818 F	GGGTTCCAATAGCGAGTTTGAT	C/T
	Cb1336818 R	TGACTGATATAACCATGCGTCAACT	
	Cb1336818_VIC_T	VIC-AACACCAGCTTTCAG-MGBNFQ	
	Cb1336818_FAM_C	6FAM-AACACCAGCTTCCAG-MGBNFQ	
Cox1910969	Cb1910969 F	ATCAGAACCTTCACTCGATTCTGC	C/T
	Cb1910969 R	AATGCTATTCGTTATGCGAAGAATG	
	Cb1910969_VIC_T	VIC-TTGAATACGAATGTTG-MGBNFQ	
	Cb1910969_FAM_C	6FAM-ATTGAATACGAACGTTG-MGBNFQ	

Part I – Precision, Limits of Quantitation, and Linearity

The precision, limits of quantitation, and linearity of both *Coxiella* assays were measured using a single plate setup for each assay. For both assays, a panel consisting of a ten-fold serial dilution series of whole-genome amplified (WGA) product from two ancestral and two derived templates were used. Eleven dilution points were made with point one having the highest DNA concentration (i.e. WGA product that would yield a Ct (cycle threshold) of ~20) and point 11 the lowest DNA concentration (i.e. $1x10^{10}$ dilution of point 1). Four assay replicates were used for each template (ancestral and derived) at every dilution point (points 1-11); 16 negative template controls were also used.

Precision

Precision, or agreement among multiple replicates of a sample, was measured for each dilution point by 1) examination of the C_T standard deviation (SD) among the four replicates at each dilution point and 2) observation of the amount of positive amplifications per dilution point. Standard deviations ranged from 0-1.82 C_Ts for both assays. Amplification of all replicates only occurred out to the 5th or 6th dilution points, depending on the assay (Figure 2). There was no amplification in negative template controls. Cross hybridization between the ancestral and derived probes was observed for the Cox1336818 assay only, but the difference in C_T between the allele-specific signal and the mismatch signal was always greater than 2.81 C_Ts and therefore large enough to provide discriminatory ability (Figure 2). The assays were precise, therefore, out to the 5th or 6th dilutions when the original template had a starting C_T of ~20. SNP genotyping calls for ancestral and derived WGA templates were 100% accurate at all dilution points which had amplification.

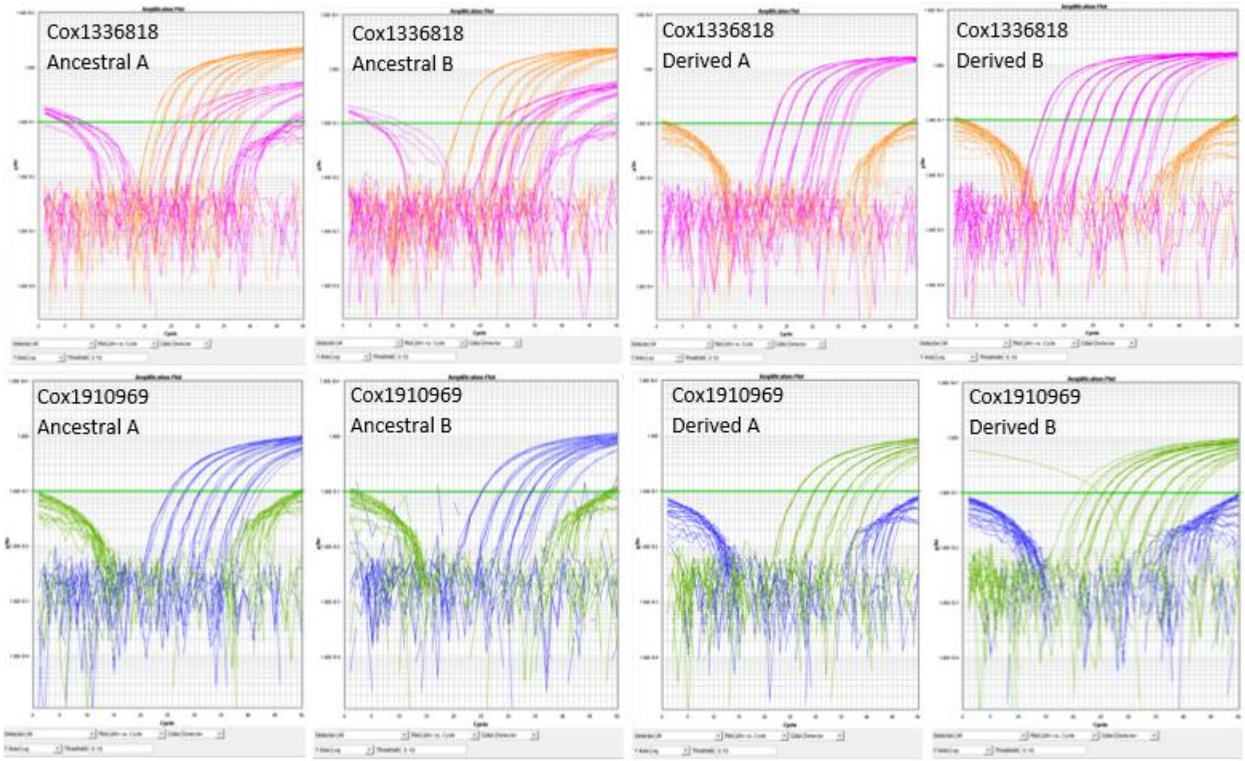

Figure 2: Precision of Cox1336818 and Cox1910969. For Cox1336818, amplification of the ancestral probe (SNP C) is shown in orange while amplification of the derived probe (SNP T) is shown in pink. For Cox1910969, amplification of the ancestral probe (SNP C) is shown in blue while amplification of the derived probe (SNP T) is shown in green.

Limits of Quantitation

Limits of quantitation, or the lowest and highest concentrations of target DNA that can be measured with reasonable precision and accuracy, were measured by looking at the amount of

correct SNP calls at each dilution point. As SNP calls were 100% accurate and there was no amplification of NTCs, the limits of quantitation were determined by the precision of each assay, which were accurate out to the 5th or 6th dilutions when the original template has a C_T of ~20. In many cases, SNP calls were also accurately made at dilutions 7-9 (approximately C_Ts of 36), with only one or two replicates of four amplifying. The upper limit of quantitation was undetermined as we did not test samples more concentrated than dilution point 1.

Linearity

Linearity, or the ability to elicit results proportional to the concentration of target DNA in the sample, was measured by plotting the average C_T across all amplifying replicates, per dilution point, per template, per assay and attributing r^2 values to the range of the linear dilutions (Figures 3 and 4). The difference in the average C_T values from one dilution point to the next ranged from 1.13 to 3.91 for both assays. The r^2 values for both assays where the linear range was determined was ≥0.9904. As we were able to determine a range where the change in C_T values per ten-fold dilution increases by ~ 3.0 C_Ts and the r^2 values in this range are ≥0.9904, we can say that each assay has a linear range. For Cox1336818, the linear range of the assay falls between ~22-37 C_Ts (Fig. 3). For Cox1910969 the linear range of the assay falls between ~26-39 C_Ts (Fig. 4).

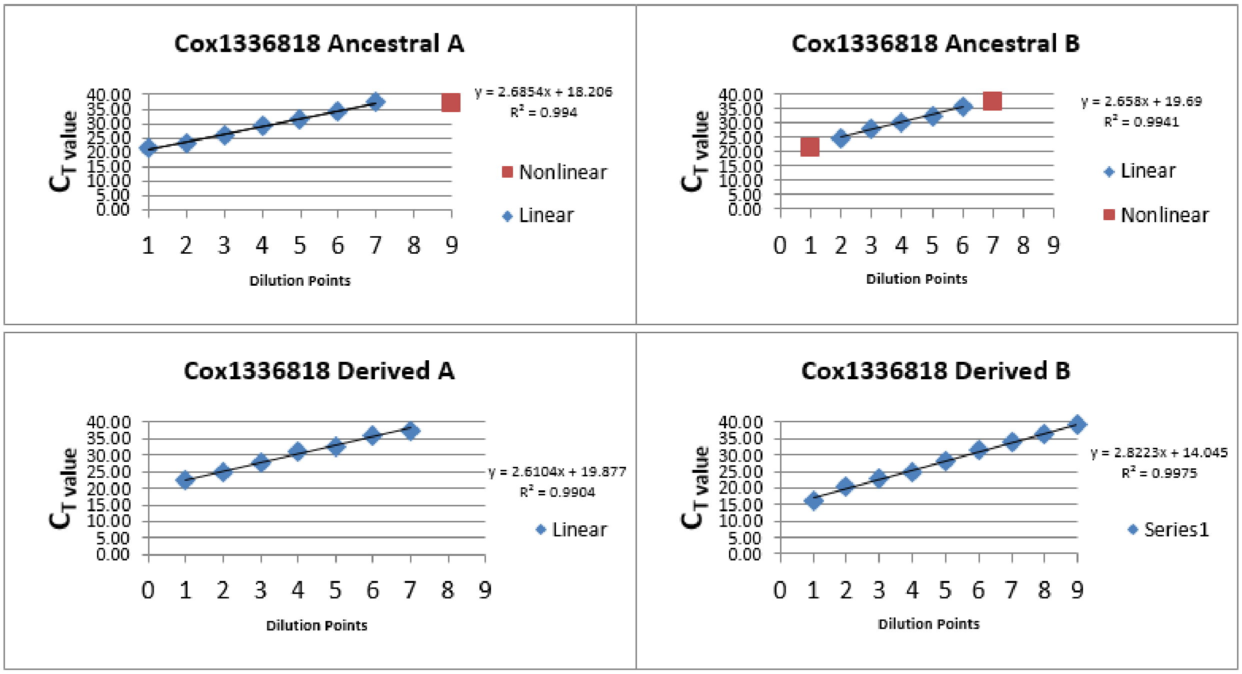

Figure 3: Plots of the average C_T value of all amplifying replicates out of four replicates, per template, per assay, for Cox1336818. For each plot, the x-axis indicates the dilution point used, (dilution 1 being the most concentrated sample and all other points being consecutive ten-fold serial dilutions from point 1); the y-axis is the average C_T value of all amplifying replicates. Linear dilution points are plotted as blue diamonds. Nonlinear replicates are plotted as red squares.

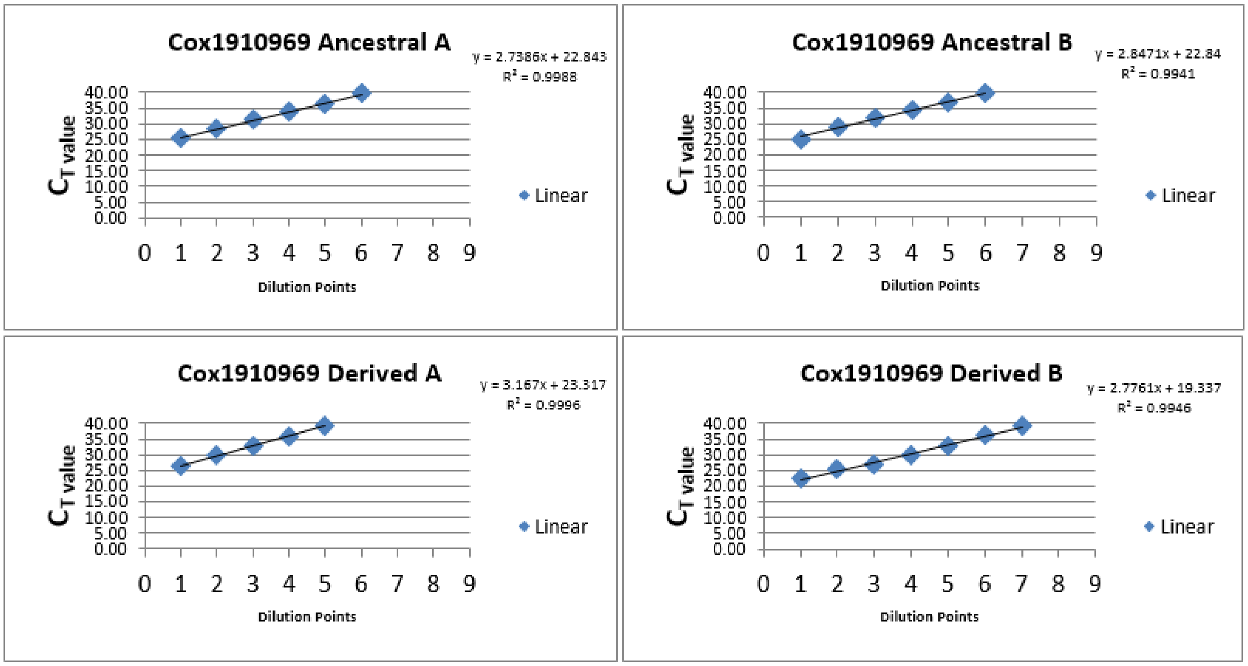

Figure 4: Plots of the average C_T value of all amplifying replicates out of four replicates, per template, per assay, for Cox1910969. For each plot, the x-axis indicates the dilution point used, (dilution 1 being the most concentrated sample and all other points being consecutive ten-fold serial dilutions from point 1); the y-axis is the average C_T value of all amplifying replicates.

Part II – Selectivity

To test selectivity, or the ability to accurately genotype in the presence of mixed samples, one ancestral and one derived WGA template (point 1 from Part I above) were used. Mixtures of ancestral:derived template were combined at ratios of 10:90, 25:75, 50:50, 75:25, and 90:10 to test the selectivity of each assay. Un-mixed samples were used as positive template controls; molecular grade water was used as a negative template control. Four replicates were tested for each control or mixture for each assay. The ΔC_T between the ancestral and derived probes was calculated for all replicates and the mixed and control samples were compared.

For both assays, the positive template controls (unmixed samples) did not show any cross hybridization of probes (Figure 5, panels "100 % Ancestral" and "100% Derived"). The mixtures had ΔC_T values ranging from 0.78 to 3.93 C_Ts. The 50:50 mixtures did not have an average ΔC_T value of zero as would be expected if they were exactly 50:50 and/or the binding efficiencies of the probes were equal (Figure 5 panels "50:50 ANC/DER"). Both assays appear to skew towards the ancestral SNP state (FAM-labeled probe for both assays) in mixtures as the 25:75 mixtures were called as ancestral despite the fact that there should have been much more derived template in the mixtures (Figure 5). This should not affect the ability of the assays to accurately detect the presence of mixtures in samples when accurate ancestral and derived controls are used to provide

a reference point when evaluating mixtures. It does, however, preclude the ability for these assays to be used to accurately estimate the ratio of mixtures in a sample.

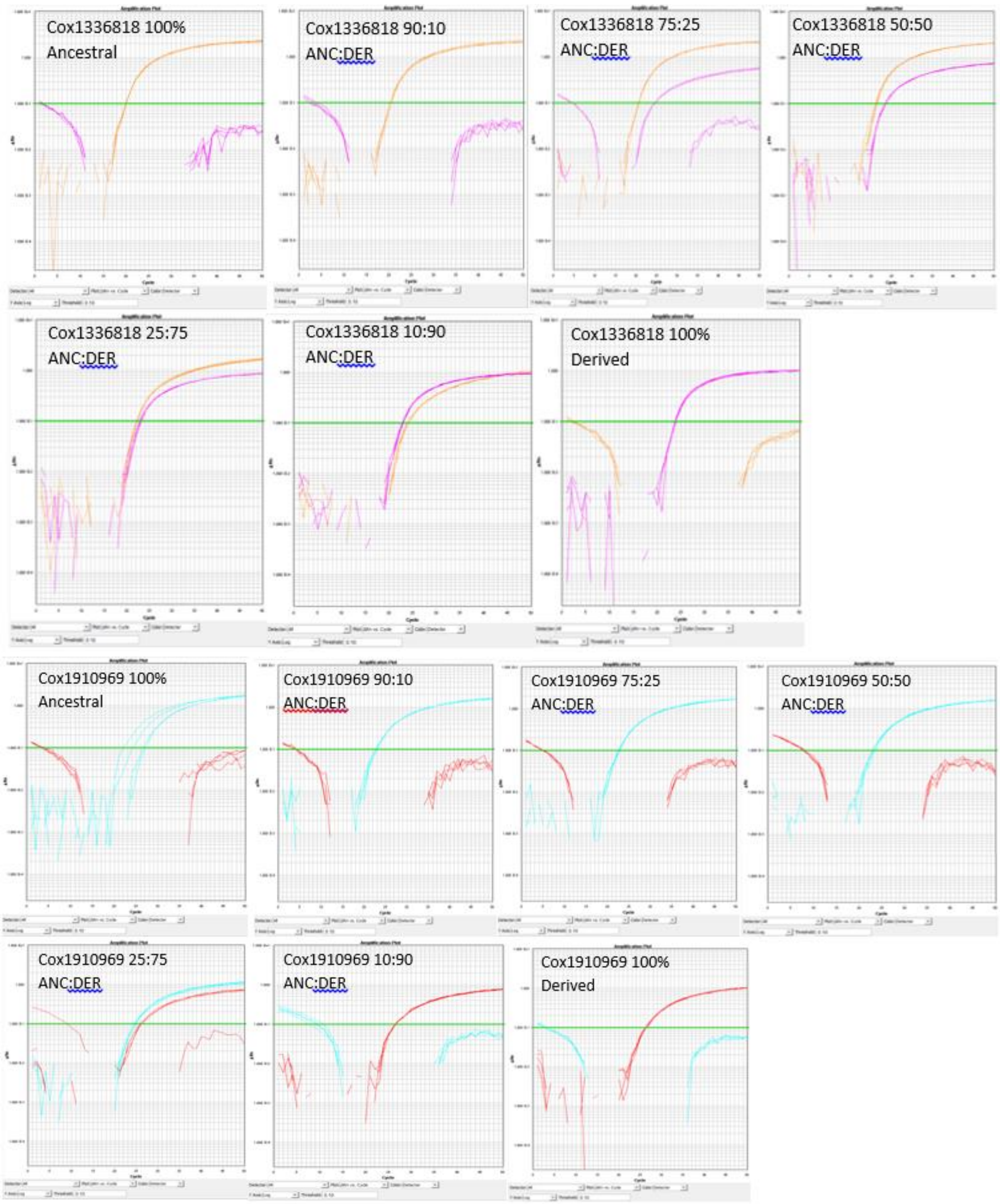

Figure 5: Selectivity of Cox1336818 and Cox1910969. Amplification plots include unmixed template and mixtures of ancestral:derived (ANC:DER) template. For Cox1336818,

amplification of the ancestral probe (SNP C, labeled with the FAM reporter) is shown in orange while amplification of the derived probe (SNP T, labeled with a VIC reporter) is shown in pink. For Cox1910969, amplification of the ancestral probe (SNP C, labeled with a FAM reporter) is shown in blue while amplification of the derived probe (SNP T, labeled with a VIC reporter) is shown in red.

Part III – Limits of Detection

The limits of detection, or the concentration at which amplification of one more replicates occurs, was measured for ancestral and derived probes in both *Coxiella* assays by testing six dilution points from Part I. The six dilution points were: two points that have successful amplification (all four replicates amplified in Part I) two points that have spotty amplification (less than four replicates amplified in Part I), and two points that failed to amplify (no amplification in Part I). Twenty-four replicates were tested for each ancestral and derived template at each of the six points selected; Twenty-four negative template controls were also tested. The points from Part I chosen for each assay per derived and ancestral templates are givin in Table 2.

The limits of detection (LOD) were measured by counting the number of replicates out of 24 which amplified for each point chosen, per template, per assay (Table 2). The LOD for both assays varied: for Cox1336818 the LOD occurred at point 7 for both probes with the ancestral probe amplifying 5/24 replicates and the derived probe amplifying 3/24 replicates (Table 2). For assay Cox1910969, the LOD varied by probe with the ancestral probe's LOD being point 6 (11/24 replicates) and the derived probes LOD = point 5 (3/24 replicates). No NTCs amplified for either assay.

Table 2: Summary of amplifications per sample for testing the Limits of Detection (LOD) for Cox1336818 and Cox1910969. For the column category: "succ" refers to dilution points where all four replicates amplified in Part I, "spotty" refers to dilution points where less than four replicates amplified in Part I and "fail" refers to dilution points where no replicates amplified in Part I. "Amp (+)" refers to the number of positive amplifications out of 24 replicates for the relevant probe/template. Red cells indicate that there was no amplification out of 24 replicates. Orange cells indicate that there were less than 24 replicates amplifying. Green cells indicate that all 24 replicates successfully amplified.

Cox1336818					Cox1910969				
Sample Genotype	Category	Dilution point from Part I	SNP Call	amp (+)	Sample Genotype	Category	Dilution point from Part I	SNP Call	amp (+)
Ancestral	fail2	11	UND	0	Ancestral	fail2	7	UND	0
Ancestral	fail1	9	UND	0	Ancestral	fail1	6	C	11
Ancestral	spotty2	7	C	5	Ancestral	spotty2	5	C	14
Ancestral	spotty1	6	C	18	Ancestral	spotty1	4	C	24
Ancestral	succ2	5	C	21	Ancestral	succ2	2	C	24
Ancestral	succ1	1	C	24	Ancestral	succ1	1	C	24
Derived	fail2	11	UND	0	Derived	fail2	7	UND	0
Derived	fail1	9	UND	0	Derived	fail1	6	UND	0
Derived	spotty2	8	UND	0	Derived	spotty2	5	T	3
Derived	spotty1	7	T	3	Derived	spotty1	4	T	15
Derived	succ2	6	T	15	Derived	succ2	2	T	22
Derived	succ1	1	T	24	Derived	succ1	1	T	24
neg	NA		UND	0	neg	NA		UND	0

Part IV - Robustness

The robustness of each assay, or the ability for the assay to amplify and genotype samples correctly at different annealing temperatures, was tested by taking three dilution points of an ancestral and derived WGA template determined from the results of Part I to be spotty (see also Part III) plus two, ten-fold dilution points after that and testing them with three different annealing temperatures. Four replicates were run for each dilution point along with eight NTCs, per assay. The annealing temperatures tested were ±5°C from 60°C and including 60°C. When tested with the standard temperature of 60°C, results were as expected (samples genotyped correctly) and little to no cross-hybridization was observed (Figure 6). When the annealing temperature was decreased to 55°C, the specificity of the probes was reduced for both assays, thus increasing cross hybridization of the probes (Figure 6) making genotyping of samples more ambiguous. When tested with an annealing temperature of 65°C, the assays failed entirely across all samples; no amplification was observed (data not shown). In summary, these assays are not very robust to changes in annealing temperature and therefore PCR should always be performed with an annealing temperature of 60°C.

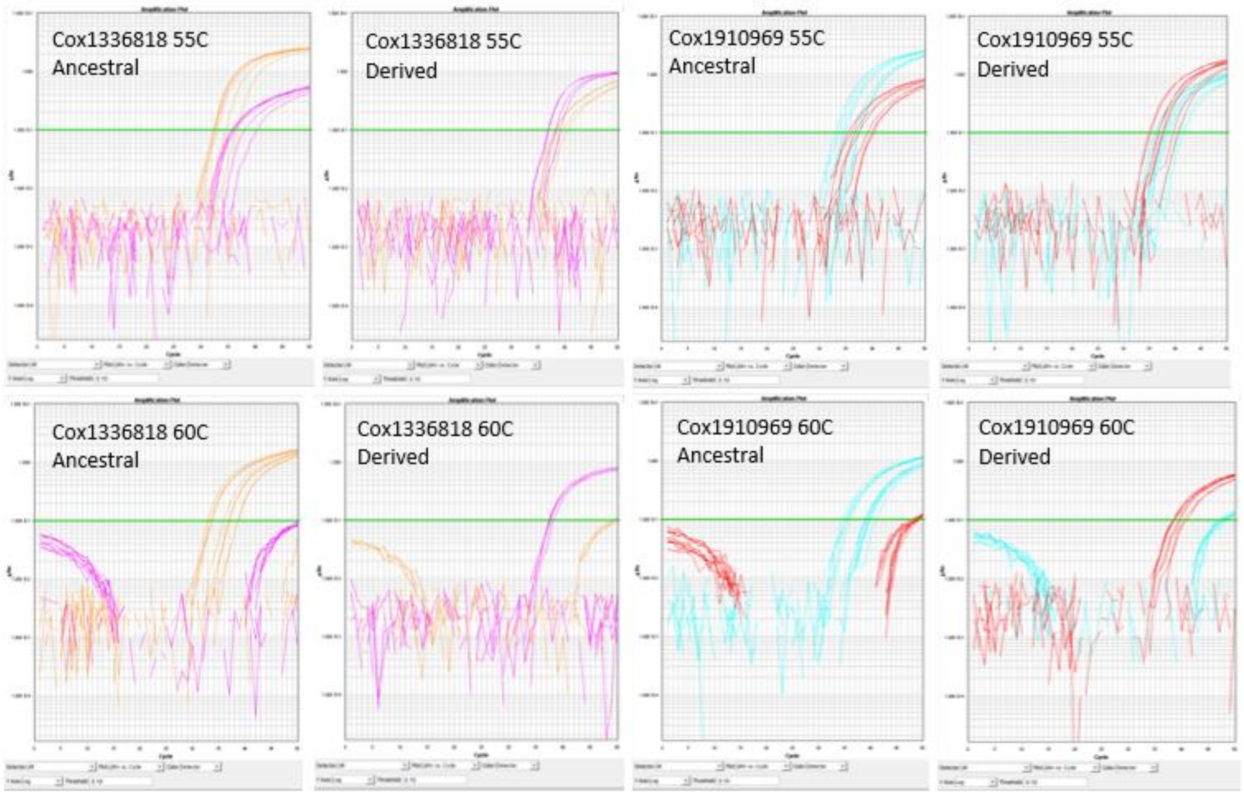

Figure 6: Robustness of the assays Cox1336818 and Cox1910969 to changes in annealing temperatures. Amplification plots include Cox1336818 and Cox1910969 at annealing temperatures of 55°C and 60°C. For Cox1336818, amplification of the ancestral probe (SNP C) is shown in orange while amplification of the derived probe (SNP T) is shown in

pink. For Cox1910969, amplification of the ancestral probe (SNP C) is shown in blue while amplification of the derived probe (SNP T) is shown in red.

Part V - IS1111 Comparison

As an additional test of these assays, a comparison between the *Coxiella*-specific detection assay, IS1111 [4], and our genotyping assays was done in order to determine the C_T value cut-off at which *Coxiella burnetii* DNA can be detected in a sample with IS1111 but no longer genotyped by our assays. For this comparison, we used all dilution points from Part I (described above) and plotted the average C_T of all amplifying replicates (per template, per probe,) along with the average C_T of the sample replicates when tested with IS1111. When running the IS1111 assay all SDS v2.4 settings were left as automatic and PCR was performed according to [4]. As shown in the plots below (Fig. 7), the last C_T value where IS1111 amplifies and where genotyping results are obtained for each assay varies from a C_T value of 29.96 to 35.65. This implies possible genotyping failures with our assay for samples that have an IS1111 C_T value ~30 or greater and likely genotyping failures with a C_T value above 35.65 (fig. 7). We would expect that genotyping failures with these two assays will increase and/or occur at a lower C_T value when the isolates being tested have a greater number of copies of IS1111 per genome. The tests below were performed using whole genome amplified product from strains CMSC1 and Q154 for the ancestral templates and ESFL1 and CMCA1c1 for the derived templates. We estimate that CMSC1, ESFL1, and CMCA1c1 have ~25 copies of IS1111, per genome and Q154 has ~46 copies, per genome (data not shown).

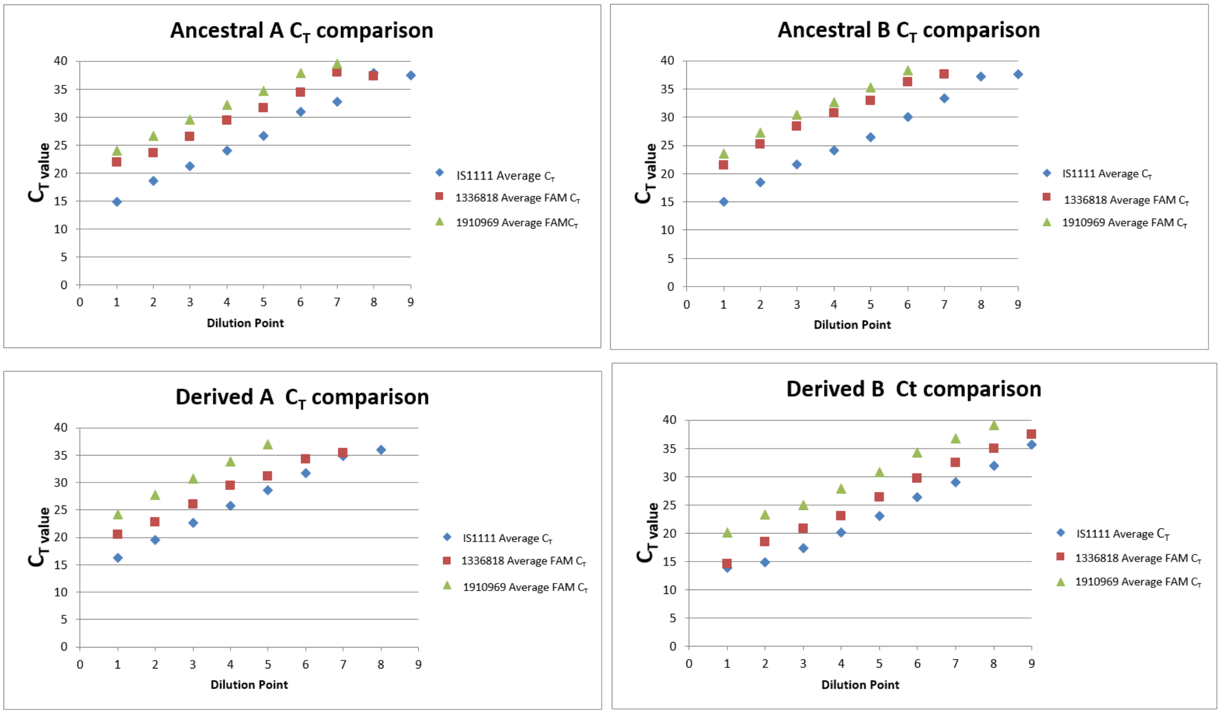

Figure 7: IS1111, Cox1336818, and Cox1910969 average C_T comparison. Each plot depicts the average C_T for all amplifying replicates across a ten-fold dilution series of either an

ancestral or derived WGA template when tested with the IS1111 [4], Cox1336818, and Cox1910969 TaqMan assays. For all plots, the blue diamonds indicate the IS1111 average C_T at each dilution point, the red squares indicate the average C_T for the probe corresponding to the sample for Cox1336818, and the green triangles indicate the average C_T for the probe corresponding to the sample for Cox1910969.

Literature Cited

1. Glazunova O, Roux V, Freylikman O, Sekeyova Z, Fournous G, Tyczka J, Tokarevich N, Kovacava E, Marrie TJ, Raoult D: ***Coxiella burnetii* genotyping**. *Emerg Infect Dis* 2005, **11**(8):1211-1217.
2. Felsenstein J: **Confidence Limits on Phylogenies: An Approach Using the Bootstrap**. *Evolution* 1985, **39**(4):783.
3. Pearson T, Hornstra HM, Hilsabeck R, Gates LT, Olivas SM, Birdsell DM, Hall CM, German S, Cook JM, Seymour ML *et al*: **High prevalence and two dominant host-specific genotypes of Coxiella burnetii in U.S. milk**. *BMC Microbiol* 2014, **14**(1):41.
4. Loftis AD, Reeves WK, Szumlas DE, Abbassy MM, Helmy IM, Moriarity JR, Dasch GA: **Rickettsial agents in Egyptian ticks collected from domestic animals**. *Exp Appl Acarol* 2006, **40**(1):67-81.
